# Prevalence and associated factors of occupational injuries among municipal solid waste collectors in four zones of Amhara region, Northwest Ethiopia

**DOI:** 10.1186/s12889-016-3483-1

**Published:** 2016-08-24

**Authors:** Debassu Eskezia, Zewdie Aderaw, Kedir Y. Ahmed, Fentaw Tadese

**Affiliations:** 1 Health Extension Program, Debre Markos Town Health Bureau, Debre Markos, Ethiopia; 2 Department of Public Health, College of Medicine and Health Science, Debre Markos University, PO Box 269, Debre Markos, Ethiopia

**Keywords:** Occupational injuries, Municipal solid waste workers

## Abstract

**Background:**

Refuse collectors are at a high risk for fatal and non-fatal occupational accidents. This is more intensified in developing countries, like Ethiopia, due to physically demanding nature of the job. However, information on occupational injuries and related factors are almost non-existent in Ethiopia. Thus, the aim of this study was to assess the prevalence of occupational injuries and its associated factors.

**Methods:**

A cross-sectional study was conducted among municipal solid waste collectors in four zones of Amhara region from February to May 2015. Computer generated simple random sampling technique was used to select the samples. Interviewer administrated questionnaires were used for the data collection process. Binary logistic regression was used to assess the association between outcome variables and explanatory variables.

**Results:**

In this study, the annual prevalence of at least one occupational injury among solid waste workers was 34.3 % (95 % CI: 29.52, 39.10). Of these, 50.7 % of them were visited health facility to receive health care. The independent predictors of at least one occupational injury were shorter service years, low monthly salary, history of job related stress, and sleeping disturbance related to the job. Being illiterate, having lower monthly income, and those who reported sleeping disturbance were significantly and positively associated with severe occupational injuries of solid waste collectors.

**Conclusion:**

The magnitude of occupational injuries among municipal solid waste collectors is lower than other similar studies conducted in Ethiopia. Based on the finding of this and other studies, job rotation among work components, improvement of employees’ income, job specific guideline regarding maximum production limits, and replacement of bags and bins with wheeled containers are an interventions expected to cope with the problem. There is also a need of specific periodic health surveillance (PHS) for refuse collectors to detect early signs of work related complaints and to monitor work ability.

## Background

Occupational injury is any physical injury conditions sustained on a worker in connection with the performance of his or her work [1]. It poses a major public health problem and are a source of substantial human and economic cost in both developed and developing countries [2, 3]. The International Labor Organization (ILO) estimates that 270 million occupational accidents and diseases occur each year, around 2.3 million workers die as a result of it [4]. Estimates from member States of the European Union indicated that the economic cost of all work-related ill health ranges from 2.6 to 3.8 % of the GDP [5, 6]. Sub-Saharan Africa countries appear to have the greatest rate of occupational injuries followed by Asia [3, 7].

Municipal solid wastes (MSW) consists of many different things including food and garden waste, paper and cardboard, glass, metals, plastics and textiles [4]. Despite waste collection has been contributing greatly to human health by reducing the risk of several infectious diseases, workers are at a high risk for fatal and non-fatal occupational accidents [8]. One systematic review showed that the occupational accident rate among Danish MSW workers was 5.6 times higher than that of total workforce [9, 10]. The standards and norms for handling municipal solid waste in developed countries have reduced its occupational health impacts substantially. However, in developing countries solid waste workers and waste pickers are at much higher risk of occupational injuries [11–15]. In these countries, the collection system is labor-intensive, workers have less protection, most waste is not safely contained in readily lift able load sizes, recycling are conducted from mixed waste, many waste pickers are children or women of child-bearing age, disposal is by open dumping, disposal equipment operators are not in closed air conditioned cabs [12, 16, 17].

In Ethiopia, municipal solid waste workers organize door to door waste collection from households and commercial areas. More typically, the waste is placed on the ground directly, thus requiring being shoveled by hand; or it is left in plastic bag or basket to be picked up by hand. Then, sack or pushcart are used to transfer the wastes to collection sites and finally it is manually emptied to a refuse truck. Therefore, the above facts indicate that workers have direct contact with solid waste, and are also exposed to strenuous working conditions. However, in Ethiopia data on health and accident consequences are almost non-existent. Two studies conducted in Addis Ababa and Northwest Ethiopia showed that the prevalence of occupational injuries among solid waste workers were 43.7 and 63.9 % respectively [11, 18]. Moreover, this study was conducted in capital cities of four zones (an administrative unit next to district) which expected to increase the representativeness of the data. Thus, the aim of this study was to assess prevalence of occupational injuries and factors associated with it.

## Methods

### Study design

A cross-sectional study design was used to assess the prevalence of occupational injuries and its associated factors among municipal solid waste collectors in four zones of Amhara region, Northwest Ethiopia.

### Study setting

The study was conducted in Debre Markos, Finote Selam, and Injibara towns and Bahir Dar city Administration from February to May 2015. Debre Markos town, Finote Selam town, Injibara town and Bahir Dar city administration are located 300, 387, 420 and 565 Km from the capital city of Ethiopia, Addis Ababa, respectively. In these towns, there are a total of 561 members of solid waste collection workers from 12 associations found in the four zone capital cities of Northwest Ethiopia.

### Participants

The source population were municipal solid waste collectors, who were working in the capital cities of four zones of Amhara region. Of these, those who had at least one year work experience and who were selected by stratified simple random sampling technique were included. Workers who had seriously illness (unable to respond due to the illnesses other than occupational injury) during the data collection period were excluded from the study.

### Sampling technique and procedure

Single population proportion formula was used to calculate the sample size of this study. By using prevalence study conducted in Northwest part of Ethiopia (*P* = 63.9 %) [11] and by assuming 95 % CI and 5 % margin of error and considering 10 % non-response rate, the final sample size was found to be 394. Stratified simple random sampling technique was used for this study. The samples selected by computer generated simple random sampling technique were distributed proportional to the number of workers in the towns.

### Data collection and data quality control

Data were collected using semi-structured interviewer administered questionnaire. The questionnaire was prepared in English and translated to Amharic and then translated back to English to check consistencies. The main contents of the questionnaire were including; socio-demographic characteristics (age, sex, residence, marital status, religion, educational status, forms of employment), working environment (working hours, PPD utilization, work experience etc.), behavioral characteristics of MSWs (sleeping disturbance (yes/no), work related stress (yes/no), Job satisfaction (yes/no), Substance (Khat, Alcohol and Cigarettes) use) and work related injuries.

Twelve diploma nurses as data collectors and two BSc Environmental health professionals as supervisors employed for the data collection process. One day training was given to data collectors, and supervisors in accordance with training manual developed beforehand. The questionnaire was pretested on 5 % MSW workers that fulfill the inclusion criteria but resided outside the study area. Inputs from the pre-test were used to modify the questionnaire in more suitable manner so as to generate the desired data. The interview was conducted in private setting and the interviewers were supervised at each site, regular meetings were held between the data collectors, supervisors and the principal investigator. Moreover, consistency was checked before, during and after entering the data in to computer.

### Operational definitions

Those who reported work related any physical damage to body tissues caused by accident or by exposure to environmental stressor in the last one year in working area were categorized as “*presence of occupational injury*”. *Severe occupational injuries* comprised of workers who reported at least one occupational injury and received health care at health facility level. *Presence of job satisfaction (yes/no)*: It is a subjective response of study participants about their job whether it is pleasurable or not. *Presence of job related stress (yes/no)*: subjective response of respondents whether they feel stress due to the job or not. *Use of personal protective device (PPD)*: A municipal solid waste collector who are using all PPD (eye goggles, boots, gloves, face shield) required for solid waste collection during collection time. *Current substance use*: those who reported use of specified substance in the last one year.

### Data processing and analysis

Coded data were organized and entered in to Epi Info version 3.5.1 computer software package and were exported to SPSS 20.0 version computer software package for further analysis. Five questionnaires found to be incomplete and excluded from the final analysis. Frequencies, percentages and means of variables were computed to describe the data. Binary logistic regression was used to assess the association between outcome variables and independent variables. To avoid unstable estimates in the subsequent model, variables that reached a *p*-value of less than 0.2 at the bivariable analysis level were kept in the final model. Finally, multivariable logistic regression model was fitted in order to identify factors associated with at least one occupational injury and severe occupational injuries of workers. *P* value of 0.05 was used to declare significant association.

## Result

### Socio-demographic characteristics

Three hundred seventy nine municipal solid waste collectors participated in the study yielding response rate of 96.2 %. The majority of them were females which account 285 (75.2 %) and the mean (±SD) age of respondents was 30.7 (±8.75) years. About 200 (52.8 %) of them were married in marital status and 159 (42 %) of them were unable to read and write in their educational status. Among respondents, 287 (75.7 %) of them reported work experience of more than 3 years (Table 1).

**Table 1:** socio-demographic characteristics of municipal solid waste collectors’ in four zones of Amhara region, Northwest Ethiopia, 2015 (*n* = 379)

Variables	Frequency	Percentage (%)
Sex		
Female	285	75.2
Male	94	24.8
Age group		
< 30 years	244	64.4
≥ 30 years	135	35.6
Residence		
Urban	363	95.8
Rural	16	4.2
Marital status		
Married	200	52.8
Single	91	24.0
Divorced	48	12.7
Widowed	25	6.6
Separated	15	4.0
Religion		
Orthodox	346	91.3
Muslim	22	5.8
Others	11	2.9
Ethnicity		
Amhara	358	94.5
Others	21	5.5
Educational status		
Can’t read and write	159	42.0
Can read and write	47	12.4
Primary schools (1–8th)	84	22.2
Secondary and above (9th +)	89	23.5
Employment pattern		
Permanent	289	76.3
Temporary	90	23.7
Monthly salary		
≥ 600 Eth. birr	307	81.0
< 600 Eth. Birr	72	19.0
Work experience		
> 3 years	287	75.7
≤ 3 years	92	24.3

### Work related and behavioral characteristics

One hundred (26.4 %) of study participants were working less than 5 days per a week. Among respondents, 332 (87.6 %) of them were used personal protective device (PPD). The reason for non-use includes; lack of PPD by 40 (85.1 %) and not comfortable with the device by 7 (14.9 %). About 261 (68.1 %) of them reported that no training was given during their employment time and 125 (33 %) and 155 (40.9) of them reported job related stress and history of sleeping disturbance in the last 1 year, respectively. Nearly one third (65.2 %), 22 (5.8 %) and 14 (3.7 %) of them had history of alcohol consumption, Khat chewing and cigarette smoking in the last 1 year respectively (Table 2).

**Table 2:** Work related and behavioral characteristics of municipal solid waste collection workers at four zones of Amhara Region, Northwest Ethiopia, 2015 (*n* = 379)

Variables	Frequency	Percentage (%)
Working hours per week		
< 5 days (39 h)	100	26.4
≥ 5 days (40 h)	279	73.6
Job related training		
Yes	118	31.1
No	261	68.9
Alcohol use		
Yes	247	65.2
No	132	34.8
Khat use		
Yes	22	5.8
No	357	94.2
Cigarette use		
Yes	14	3.7
No	365	96.3
Job satisfaction		
Yes	236	62.3
No	143	37.7
PPD use		
Yes	332	87.6
No	47	12.4
Reason for non-use		
Lack of PPD	40	85.1
Not comfortable	7	14.9
Sleeping disturbance		
Yes	155	40.9
No	224	59.1
Job related stress		
Yes	125	33.0
No	254	67.0

*PPD* personal protective devise

### Prevalence of occupational injuries

Out of 379 municipal solid waste collection workers, 130 (34.3 %) with 95 % CI (29.52, 39.10) were reported at least one injury in the last one year. Of these, 54 (41.5 %) workers were injured once and the rest 76 (58.5 %) of them injured two or more times. About 45 (34.6) of them reported injured body part of hand only. The most common type of injury was cut/punctures which was 68 (52.3 %) followed by abrasion 20 (15.40 %) and dislocation 12 (9.23 %). Regarding the source of injury, 47 (37.0 %) of them hit by falling objects and 29 (22.0 %) of them injured by hand tools. Ninety six (73.8 %) of them were lost more than ten working days due to an injury. Of those who reported occupational injury, 66 (50.7 %) treated at health facility level, of which 13 (10 %) of them admitted in Hospital (Table 3).

**Table 3:** Prevalence of occupational injuries among municipal solid waste collection workers at four zones of Amhara Region, Northwest Ethiopia, 2015

Variables	Frequency	Percentage (%)
Occupational injury		
Yes	130	34.3
No	249	65.7
Frequency of occupational injury (*n* = 130)		
Once	54	41.5
Two or more times	76	58.5
Types of injury		
Cut/Puncture	68	52.30
Abrasion	20	15.40
Dislocation	12	9.23
Fracture	11	8.46
Ear injury	10	7.69
Eye injury	9	6.92
Parts of body injured		
Hand	45	34.6
Leg	26	20.0
above neck	21	16.2
more than one body parts	38	29.2
Source of injury		
Hit by falling objects	47	37.0
Hand tools	29	22.0
Falls	24	18.0
Lifting heavy objects	13	10.0
Splintering objects	12	9.2
Collision	5	3.8
Working days lost		
< 10 days	34	26.2
≥ 10 days	96	73.8
Treated at health facility		
Yes	66	50.7
No	64	49.3
Admission due to an injury		
Yes	13	10
No	117	90

### Factors associated with occupational injuries

In multivariable analysis, work experience, monthly salary, sleeping disturbance, and job related stress were significantly associated with at least one occupational injury of municipal solid collection workers.

The likelihood of occupational injury was found to be significantly higher (AOR = 1.92 95 % CI: 1.11, 3.31) among respondents with three or less service years. Having monthly salary of less than 600 Eth. Birr (AOR = 3.0 95 % CI: 1.64, 5.48) was significantly and positively associated with occupational injury. The odds of occupational injury was 2.57 times (95 % CI: 1.48, 4.47) higher among those who reported sleeping disturbance as compared to their counterparts. Job related stress (AOR = 1.94 95 % CI: 1.11, 3.40) was also significantly and positively associated with occupational injury (Table 4).

**Table 4:** Factors associated with occupational injuries among municipal solid waste collection workers at four zones of Amhara Region, Northwest Ethiopia, 2015

Variables	Occupational injuries		Crude Odd Ratio with 95 % CI	Adjusted Odd Ratio with 95 % CI
	Yes	No		
Work experience				
≤ 3 years	43	49	2.02 (1.25–3.26)	1.92 (1.11, 3.31)*
> 3 years	87	200	1	1
Residence				
Urban	128	135	3.81 (0.85, 17.04)	4.43 (0.9, 21.83)
Rural	2	14	1	1
Monthly salary				
< 600 Eth. Birr	46	26	4.70 (2.73, 8.08)	3.0 (1.64, 5.48)**
≥ 600 Eth. Birr	84	223	1	1
Sleeping disturbance				
Yes	85	70	4.83 (3.07, 7.61)	2.57 (1.48, 4.47)**
No	45	179	1	1
Job related stress				
Yes	70	55	4.12 (2.61, 6.50)	1.94 (1.11, 3.40)*
No	60	194	1	1

Note: 1 = Reference ** = *p* ≤ 0.001 * = *p* < 0.05

From all variables entered in the final multivariable model fitted for severe occupational injuries, sleeping disturbance related to the job, monthly salary, and literacy level were remained significant after adjusting for other independent factors. Monthly salary (AOR = 4.09 95 % CI: 2.15, 7.76) and literacy level (AOR = 2.22 95 % CI: 1.22, 4.04) were socio-demographic variables significantly associated with severe occupational injuries. Job related sleeping disturbance (AOR = 2.24 95 % CI: 1.22, 4.11) was also another variable that showed significant association with severe occupational injuries (Table 5).

**Table 5:** Factors associated with severe occupational injuries among municipal solid waste collection workers at four zones of Amhara Region, Northwest Ethiopia, 2015

Variables	Severe Occupational injuries		Crude Odds Ratio with 95 % CI	Adjusted Odds Ratio with 95 % CI
	Yes	No		
Sleeping disturbance				
Yes	42	113	3.10 (1.78, 5.38)	2.24 (1.22, 4.11)*
No	200	24	1	1
Monthly salary				
< 600 Eth Birr	29	43	4.92 (2.75, 8.82)	4.09 (2.15, 7.76)**
≥ 600 Eth. Birr	37	270	1	1
Literacy level				
Illiterate	43	163	1.72 (0.99, 2.99)	2.22 (1.22, 4.04)*
literate	23	150	1	1

Note: 1 = Reference ** = *p* ≤ 0.001 * = *p* < 0.05

## Discussion

This study showed that the overall annual prevalence of occupational injury was found to be 34.3 %. The result of this study found to be lower than studies conducted in Addis Ababa (43.7 %) and Northwest Ethiopia (63.9 %) [11, 18]. As municipal Solid waste is produced as a result of economic productivity and consumption; the compositions of wastes collected from small towns expected to be less hazardous and more garbage in nature and smaller in size which could explain the above difference. More than half of respondents also reported occupational injury of more than once in the last one year. In this study, cut/puncture, abrasion and dislocation are the most common type of injuries reported. Similarly, different local and abroad studies reported that cut and/or puncture as the most common type of occupational injuries [4, 11, 16, 18, 19].

Regarding the body parts injured, hands are the most common body parts followed leg. The same finding was reported in Ethiopian studies conducted among solid waste collectors [11, 18]. The possible explanation for this may be due to the fact that waste collectors wipe waste and put it in to the cart and tracks using their hands which increase the probability of having injury [18]. In this study, hit by falling objects and hand tools are the most common patterns of injuries reported. Similarly, different literatures are showing the same regarding common agents of work related injuries [4, 12].

The occurrence of any type of (either non-severe or severe) occupational injuries are significantly associated with monthly salary of the workers. This is explained by the fact that better salaries mean better chances of treatment and better protection from work related accidents which helps them to be less exposure to waste dust and less contact with waste material [20]. Having work experience of three or less years was positively associated with at least one occupational injury. This is in line with already existing knowledge that more experienced waste collectors work safer [21–24]. The possible explanation might be due to both life experience and years of experience on the job better predict job performance [12]. The higher level of job dissatisfaction and social stigma in new workers owing to the nature of job might also explain the finding. In contrary, other studies conducted globally showed that more experienced waste collectors are more vulnerable to occupational accidents [4, 11, 16, 19].

Having job related stress showed significant association with occupational injury of solid waste collectors. This finding is in line with other studies conducted among industrial workers [1, 25, 26]. The possible explanation for this finding could be workers who had stress might be preoccupied by extra thinking which emanated from physical symptoms (which includes headache and abdominal pain), and disturbance of psychological and family relationships related to stress. The result of the study has revealed that the occurrence of any type of occupational injuries are significantly related to sleeping disturbance. This finding is similar with other studies conducted in Ethiopia [11, 18]. A meta-analysis also suggested that workers with sleep problems had a 1.62 times higher risk of being injured at work compared to workers without sleep problems [27]. As more than three-fourth of study participants were women, the increased association might be due to factors such as marital status, number of children in the house and family responsibilities that affect the quality of life and sleeping pattern of women. Sleeping disturbance also affect the ability to maintain wakefulness, concentration, ability in assessing or watching the work environment and working conditions. Moreover, almost all refuse collection works are conducted at the morning time in Ethiopian setting which might also be the possible explanation.

The literacy level of workers was significantly associated with the occurrence of severe occupational injuries. This might be due to the fact that education is more likely to increase workers safety and health practice that can prevent them from occupational injuries [1].

The use of the capital cities of four zones which increase the representativeness of the information is the strength of the study. Unable to infer causal relationship owing to cross-sectional nature of the study and the presence of recall bias due to the long time pass are among the potential limitations.

## Conclusion

This study demonstrated that the magnitude of occupational injuries among municipal solid waste collectors found to be lower compared to similar studies conducted in Ethiopia. Working for short service years, having lower monthly income, being stressed due to the job and experiencing sleep disturbance are significantly and positively associated with at least one occupational injury. Moreover, being illiterate, having lower monthly income and those who reported sleeping disturbance had significant association with severe occupational injuries of solid waste collectors Based on the finding of this and other studies, job rotation among work components, improvement of employees’ income, job specific guideline regarding maximum production limits, and replacement of bags and bins with wheeled containers are an interventions expected to cope with the problem. There is also a need of specific periodic health surveillance (PHS) for refuse collectors to detect early signs of work related complaints and to monitor work ability. In addition, the relationship between work experience and occupational injuries need further investigation.
